# Variation in pediatric egg and milk allergy management: A multinational survey in Ireland, Spain, and Canada

**DOI:** 10.1111/pai.70301

**Published:** 2026-02-11

**Authors:** Aoife Gallagher, Jennifer L. P. Protudjer, Douglas P. Mack, María de los Angeles Gonzalez Labrador, Cristina Muñoz, Caoimhe Cronin, Juan Trujillo

**Affiliations:** ^1^ Department of Paediatrics and Child Health University College Cork Cork Ireland; ^2^ Department of Pediatrics and Child Health University of Manitoba Winnipeg Manitoba Canada; ^3^ Department of Pediatrics McMaster University Hamilton Ontario Canada; ^4^ Hospital Universitario de Salamanca Salamanca Spain; ^5^ Hospital Universitario Severo Ochoa Madrid Spain; ^6^ Cork University Hospital Cork Ireland; ^7^ Department of Paediatrics and Child Health Cork University Hospital, University College Cork, Irish Centre for Maternal and Child Health Research (INFANT), HRB Clinical Research Facility Cork (CRF‐C) Cork Ireland

**Keywords:** egg allergy, food allergy management, food ladder, international survey, milk allergy, oral immunotherapy, pediatric allergy

To the Editor,

Egg and milk allergies are some of the most common food allergies in children and the main causes of anaphylaxis in the first 2 years of life^1^ Food allergy is also a significant cause of other adverse medical and psychological outcomes, with effects on nutrition and growth as well as quality of life for children and their families.^2^ Despite their frequency, there is no international consensus on the best approach to diagnosis and management.^3^ Approaches range from strict avoidance, through structured dietary advancement via food ladders, to oral immunotherapy (OIT).^4^, ^5^ While all strategies have been reported in the literature, the extent to which they are adopted in real‐world clinical practice varies.

To better understand current international practice, we conducted a multinational survey of pediatric allergy clinicians in Ireland (*n* = 19), Canada (*n* = 22), and Spain (*n* = 67). The questionnaire was jointly developed by pediatric allergy experts from the three countries, and refined through iterative review to ensure clinical relevance and clarity across all settings. Participants were recruited through national allergy society mailing lists in 2024–2025. The invitation was sent to approximately 20 clinicians in Ireland, 250 in Canada, and 950 in Spain, giving response rates of 95%, 8%, and 7% respectively. Participants included allergists, pediatric allergists, immunologists, pediatricians with a special interest in allergy, as well as allergy and pediatric trainee physicians. Stratified sampling was not used in the recruitment process. This descriptive survey was not designed for inferential analysis so no formal statistical testing was performed. The survey addressed practice settings, diagnostic methods, management approaches, follow‐up, and telemedicine use. This study was approved by ethics boards in each of the included countries. Importantly, the survey was intended to describe reported clinician practices within participating samples rather than to infer or compare national practice patterns, and any observed variation should be interpreted in this descriptive context.

Practice settings and clinician expertise varied markedly. Irish respondents represented a spectrum of tertiary hospitals, regional centres, and community practice, reflecting a distributed model of care. In contrast, Canadian respondents were concentrated in private and tertiary‐care centres, with respondents almost exclusively allergy specialists, reflecting a high degree of subspecialisation. Spanish respondents were largely hospital‐based, split between pediatric and adult allergists. These observations suggest that clinician‐reported practice settings may be shaped by underlying healthcare structures. Ireland's limited specialist numbers necessitate an integrated model spanning community and hospital care, whereas Canadian practice is highly subspecialised. Spanish care remains largely hospital centred (Figure 1).

**FIGURE 1:**
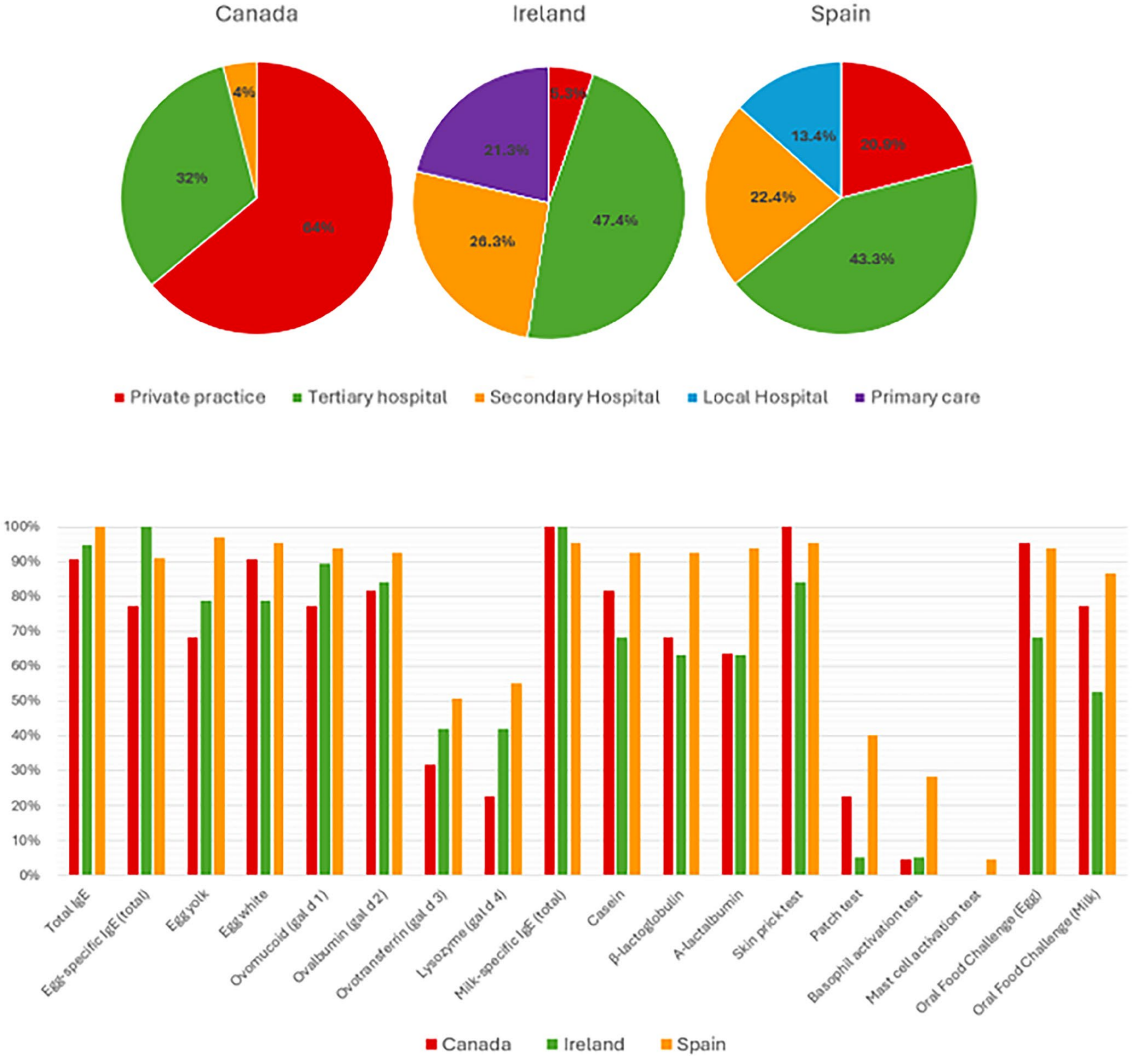
Practice settings and available diagnostics.

Diagnostic approaches reported by respondents also diverged. Oral food challenges were reported as widely available in Spain (94% for egg, 87% for milk) and Canada (96%, 77%, respectively) but less so in Ireland (68%, 53%, respectively). One in five Irish clinicians reported diagnosing egg or milk allergy based on history alone, without confirmatory testing. Component‐resolved diagnostics were reported to be most comprehensive in Spain, including assays for minor allergens (ovomucoid, ovalbumin, α‐lactalbumin, β‐lactoglobulin, κ‐casein). Canadian and Irish respondents focused on the major components (total IgE and Specific IgE to whole egg) (Figure 1). These discrepancies likely reflect resource allocation: food challenges and advanced diagnostics are expensive and time‐consuming, but without them, the risk of overdiagnosis or misclassification increases.

Management strategies showed the sharpest contrasts across respondents. In Ireland, virtually all clinicians reported using the Irish Food Allergy Network (IFAN) egg ladder (95%) and International Milk Allergy in Primary Care (iMAP) milk ladder (95%) as standard of care, with no centres offering OIT. This reflects a nationally coordinated approach, where ladders have become embedded in clinical practice. In Canada, respondent‐reported practice was more varied: 32% recommended strict avoidance, but most used ladders (77%) and 41% offered OIT, sometimes in combination with omalizumab (13.6%). In Spain, respondents most frequently reported strict avoidance as their management of choice (71% for egg, 76% for milk), yet over half of respondents reported offering OIT (53% egg, 63% milk), with almost one quarter using OIT with omalizumab (22.7% egg, 26.9% milk). Thus, Ireland exemplifies a ladder‐driven approach, Spanish respondents an avoidance‐plus‐OIT model, and Canadian respondents a hybrid (Table 1).

**TABLE 1:** Summary of reported available diagnostics and management strategies.

	Canada (%)	Ireland (%)	Spain (%)
Diagnostics			
Total IgE	90.9	94.7	100
Skin prick test	100	84.2	95.5
Egg			
Specific IgE	77.3	100	91
Component‐resolved diagnostics	22.7–81.8	42.1–89.5	50.7–95.5
OFC	95.5	68.4	94
Milk			
Specific IgE	100	100	95.5
Component‐resolved diagnostics	63.6–81.8	63.2–68.4	92.5–94
OFC	77.3	52.6	86.6
Management			
Egg			
Avoidance	31.8	10.5	71.2
Ladders	77.3	94.7	32.3
OIT	40.9	0	53
OIT with omalizumab	13.6	0	22.7
Milk			
Avoidance	50	10.5	76.1
Ladders	77.3	94.7	32.3
OIT	50	0	62.7
OIT with omalizumab	13.6	0	26.9

We further explored the respondent‐reported types of OIT used in Canada and Spain. Canadian clinicians most often reported initiating egg OIT with raw, pasteurized, or lyophilised egg, (56.3%), while smaller proportions used baked egg first (18.9%) or tailored their choice to specific IgE levels (12.6%). For milk OIT, raw milk was most common (40%), with some use of baked milk followed by raw (20%), or Ultra‐Heat Treated (UHT) formulations (13.4%). In Spain, respondents more often reported individualized approaches: over half of clinicians reported selecting OIT type according to egg‐ or milk‐specific IgE results (57.1% egg, 50.8% milk). Some began with baked egg (14.3%) or baked milk (10.2%) before progressing to raw, while others used UHT milk protocols (11.9%). These findings underscore both the diversity of OIT practice and the absence of a unified evidence‐based pathway across countries (Figure 2).

**FIGURE 2:**
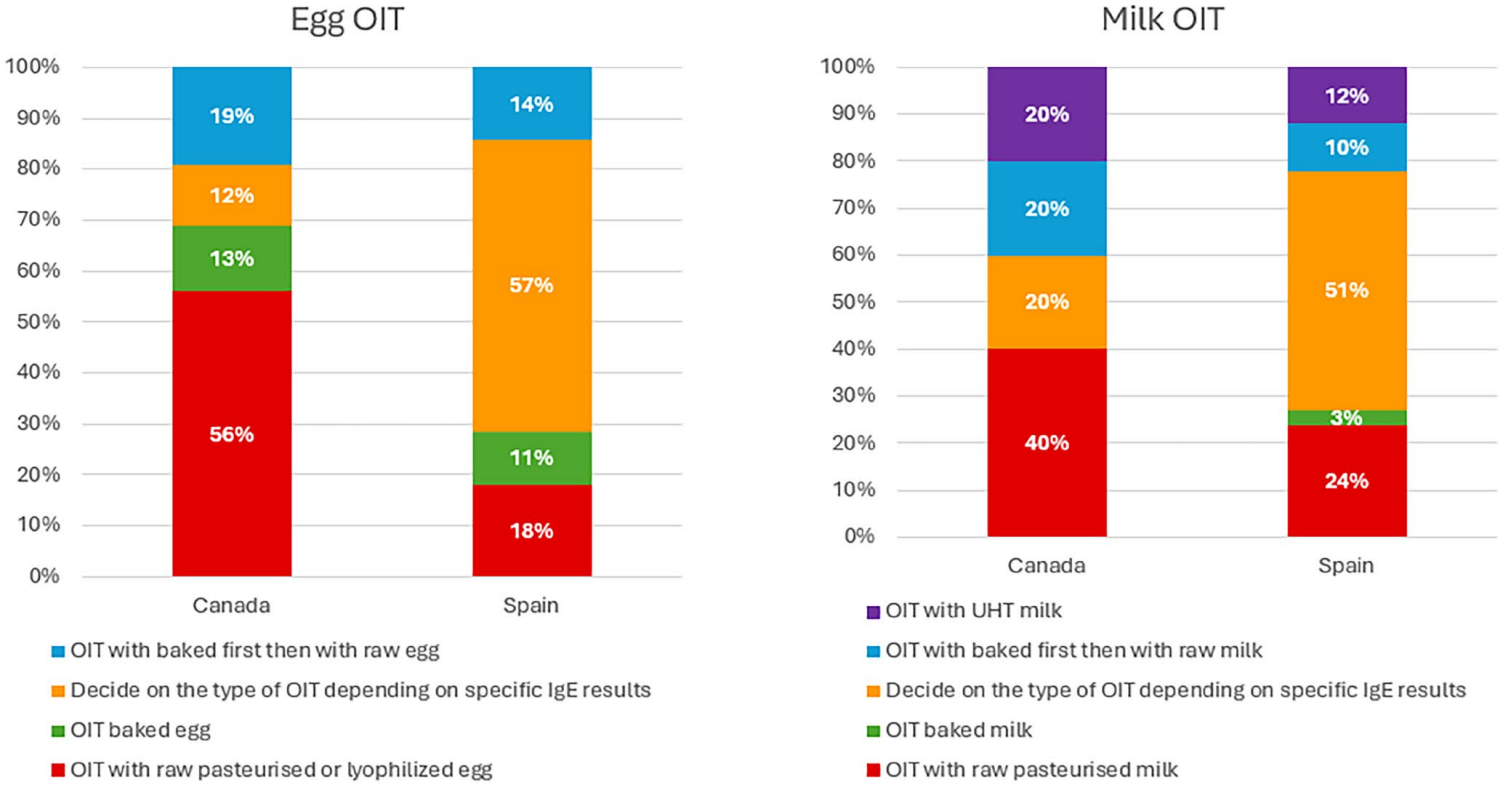
Oral immunotherapy (OIT) approaches for egg and milk allergy in Canada and Spain.

Follow‐up practices also varied across respondents. Spanish clinicians reported the most intensive review schedules, with some patients on ladders or OIT being seen weekly or fortnightly. By contrast, Canadian and Irish respondents reported their practices tended toward less frequent follow‐up, often at three‐ to six‐month intervals. This difference likely reflects both resource availability and risk tolerance: OIT, in particular, requires close monitoring for safety, whereas ladder‐based approaches are often home‐managed.

Telemedicine has emerged as a critical modality since the COVID‐19 pandemic.^6^ In our survey, 86% of Canadian and 84% of Irish clinicians reported using telemedicine when treating patients with egg and/or milk allergy, compared with 57% in Spain. In all countries, however, virtual visits were reported to account for fewer than 25% of encounters, suggesting telemedicine remains an adjunct rather than a replacement for in‐person care among respondents. Nonetheless, its potential role in reducing patient burden and extending specialist access is clear, especially in countries like Ireland where families often travel long distances to allergy centres.

Taken together, these findings illustrate how national context shapes pediatric food allergy care. Many of the reported differences likely reflect broader system‐level factors, including healthcare infrastructure, funding models, and resource availability. These variations have the potential to affect patient safety, access and quality of life. Ireland's reliance on ladders represents a structured, low‐cost, standardized approach that provides families with a clear pathway. Canadian respondents reported a hybrid model reflecting both high levels of subspecialisation and emerging adoption of OIT. Variation in physician compensation models across Canadian provinces, with some relying predominantly on fee‐for‐service schedules, while others are increasingly adopting blended or alternative payment models may contribute to regional differences in clinical practice,^7^ although our study did not collect province‐level data to explore this further. Spanish respondents demonstrated both conservatism, with a continued emphasis on avoidance, and innovation, with extensive OIT programs.

These differences have important implications. From a family perspective, the burden of treatment varies greatly depending on whether a child is managed by avoidance (with its social and dietary restrictions^8^), ladders (requiring structured home reintroduction^8^, ^9^), or OIT (demanding intensive clinic follow‐up and risk management). From a systems perspective, the resource requirements of OIT,^5^ including staffing, facilities, and emergency preparedness, are substantial compared to ladders or avoidance.

Our findings also illustrate important opportunities. Greater access to oral food challenges could enhance diagnostic accuracy in some settings. The growing use of OIT highlights the need for standardized protocols to ensure consistency and comparability across centres. The diversity of approaches underscores the need for comparative effectiveness research: prospective trials and registries comparing ladders, avoidance, and OIT in matched populations, measuring not only desensitization and sustained unresponsiveness but also safety, quality of life, and cost‐effectiveness.

This study has several limitations. Recruitment through national allergy society mailing lists may introduce selection bias, and the absence of stratified sampling resulted in differing distributions of practice settings across countries. As with all survey research, response bias is possible, and the overall sample size, particularly in Spain and Canada, limits precision. Consequently, the findings should be interpreted as descriptive of the clinicians who responded rather than fully generalisable to national practice.

In conclusion, this multinational survey documents international variation in the clinician‐reported approaches to the diagnosis and management of pediatric egg and milk allergy. While Irish respondents have standardized around ladder‐based reintroduction, Spanish respondents have embraced OIT, and Canadian respondents blend multiple approaches. Harmonized, evidence‐based guidelines are urgently needed to provide clarity for clinicians and families, reduce unwarranted variation, and ensure equitable access to safe and effective care. International collaboration will be essential to achieve this goal.

## AUTHOR CONTRIBUTIONS

**Aoife Gallagher:** Conceptualization; investigation; writing – original draft; writing – review and editing; methodology; formal analysis; project administration; data curation. **Jennifer L. P. Protudjer:** Conceptualization; writing – review and editing; supervision; data curation. **Douglas P. Mack:** Conceptualization; data curation; writing – review and editing. **María de los Angeles Gonzalez Labrador:** Writing – review and editing; data curation. **Cristina Muñoz:** Writing – review and editing. **Caoimhe Cronin:** Writing – review and editing; conceptualization. **Juan Trujillo:** Conceptualization; writing – review and editing; supervision.

## CONFLICT OF INTEREST STATEMENT

The authors declare that they have no conflicts of interest relevant to this work.
